# Contextual Factors Affecting Continuity of Follow-Up Care After Hospital Discharge for Patients with Chronic Diseases in Sudan: A Qualitative Study with Causal Loop Diagram Insights

**DOI:** 10.1177/11786329251349916

**Published:** 2025-06-24

**Authors:** Asma MohamedSharif, Armin Gemperli

**Affiliations:** 1 Faculty of Health Sciences and Medicine, University of Lucerne, Switzerland; 2 Center of Primary and Community Care, University of Lucerne, Switzerland

**Keywords:** system thinking, transitional care, integrated care, healthcare system, low- and middle-income countries, noncommunicable diseases

## Abstract

This study aims to identify factors influencing the continuity of follow-up care after hospital discharge from the perspectives of physicians and key healthcare stakeholders and map their interactions to facilitate understanding of dynamic relationships. We conducted audio-recorded semi-structured interviews with 17 participants (10 medical doctors, 3 state key informants, and 4 federal key informants) in Khartoum State, Sudan. Data analysis included thematic analysis to identify the factors and purposive text analysis to develop a causal loop diagram. We identified 39 factors affecting the continuity of follow-up care from hospital to home, categorized into 5 challenges: follow-up care adherence, quality of pre-discharge patient education, efficiency of the referral system, primary healthcare center accessibility, and quality improvement efforts. The study identified 2 balancing loops and 5 reinforcement feedback loops affecting follow-up care post-hospital discharge. The low adherence to follow-up care proposes quality improvement efforts as a solution, however, the high workload, resource depletion, referral system inefficiency, and quality improvement stagnation reinforcing loops impede progress in this direction. We recommend enhancing pre-discharge patient education and using multisectoral approaches to improve primary healthcare, optimize referrals with digital tools, and address staff turnover, to strengthen follow-up care.

## Introduction

Chronic diseases are especially challenging to treat in low- and middle-income countries (LMICs), where they are a pressing public health concern. Chronic diseases are long-term health conditions that persist over time. Common examples include cardiovascular diseases, diabetes, chronic respiratory diseases, cancer, chronic kidney disease, and neurodegenerative disorders such as Alzheimer’s disease.^1^ In Sudan, chronic diseases account for about 52% of all deaths.^2^ Care of patients with chronic diseases often ceases after hospital discharge,^3^ and they are at higher risk of hospital readmission and emergency department (ED) visits.^4^ Hospital readmission often is a result of a lack of follow-up care in the community and limited access to high-quality services.^5,6^ A systematic review from global evidence showed that most hospital readmissions and deaths could be prevented.^7^ Health systems could better address the needs of patients with chronic conditions in LMICs,^8,9^ however the health systems in most low-income countries are unprepared, or non-responsive to combat the threat of chronic conditions.^8,9^

Sudan’s healthcare delivery system is structured in 3 tiers: The Federal Ministry of Health (FMOH), state health ministries, and local health management authorities. Healthcare services are delivered at 3 levels: the highest level consists of teaching, general, and specialist hospitals that offer secondary and tertiary care. The intermediate level comprises rural hospitals that provide secondary care and diagnostic services. The primary level offers care through various outlets, including primary healthcare units, dressing stations, dispensaries, and health centers.^10^ In resource-constrained health systems such as Sudan, healthcare is fragmented, with geographic disparities, uncoordinated providers, inconsistent health insurance coverage, and financial barriers, resulting in uneven access and variable quality of care.^11,12^ Uneven access and variable quality of care manifest as urban–rural disparities. Healthcare facilities and staff are concentrated in cities like Khartoum, while rural areas face severe shortages of resources and trained personnel. Inconsistent policy implementation and uneven health financing further contribute to variability in service quality across regions.^11^ In Sudan, financial coverage for healthcare services involves a mix of public funding, private insurance, and out-of-pocket payments, with the National Health Insurance Fund (NHIF) playing a significant role.^13^ The NHIF provides free follow-up visits at primary healthcare facilities and covers 75% of medication costs for insured patients. However, the scheme only covers approximately 37.3% of the population, limiting its overall impact on equitable access to follow-up care and hospital discharge continuity.^14^ Healthcare services include both public institutions, such as general and teaching hospitals and primary healthcare units, and private entities, including hospitals and specialized clinics. Access to health services in principle follows a gatekeeping system with primary care providers serving as the first point of contact and referrals are required for secondary and tertiary care, although emergency services can be accessed directly.^15^

Health programs often invest in short-sighted policies and experience political resistance in the face of complexity.^16^ It is important to consider the local context of transitional care interventions, the factors that influence their implementation,^17^ and the dynamics of these factors before designing such interventions. Causal Loop Diagram (CLD) is a common systems dynamics tool that produces qualitative illustrations of mental models, focusing on highlighting causality and feedback loops, and has been widely used in health services research.^18-20^ The CLD enables the identification of leverage points, which are the factors within the CLD where change is likely to result in significant shifts elsewhere in the system.^21^

While a separate study has explored the continuity of care from the patient perspective,^22^ there is no published research specifically examining continuity of follow-up care after hospital discharge from the perspectives of physicians and key healthcare stakeholders. The objective of this study is to examine the contextual factors that affect the continuity of follow-up care after hospital discharge from the perspectives of physicians and key healthcare stakeholders. We aimed to identify the main factors influencing follow-up care after hospital discharge and map the interactions and feedback loops among these factors to understand the dynamic relationships between those factors.

## Methods

### Study Design

The study followed a qualitative design utilizing semi-structured interviews with medical doctors and stakeholders, followed by thematic and purposive text analysis to identify contextual factors of continuity of transitional care. A causal loop diagram was developed to visually represent the causal relationships between the contextual factors. We used the Consolidated Criteria for Reporting Qualitative Research (COREQ) checklist to guide reporting^23^ (Supplemental S1 File: COREQ checklist).

### Study Setting and Participants

This study was conducted in Sudan, the recruitment started in July 2022 and ended in September 2022. The study participants were medical doctors at the Ibrahim Malik Teaching Hospital and the Omar Ibn Al Khattab Primary Healthcare Center. The Ibrahim Malik Teaching Hospital offers a 24/7 emergency service and multiple outpatient clinics. It has 8 departments and provides health services to all age groups. The hospital is located in Alsahafa, Khartoum locality. We interviewed medical doctors who worked in the Department of Internal Medicine. The Department of Internal Medicine comprises a 44-bed short-stay ward and a 30-bed long-stay ward. The Omar Ibn Al Khattab Primary Healthcare Center, located in Arquit, provides the standard primary healthcare package and serves the population living in Arquit and Alsahafa. Additionally, the study involved stakeholders (directors) from the Federal Ministry of Health and the Khartoum State Ministry of Health. We used convenience sampling to recruit medical doctors and purposive sampling to recruit state and federal stakeholders. The sample size was determined using the principle of data saturation. During the interviews, participants were asked to identify stakeholders they considered involved in the discharge process and improvement. Once the same individuals and roles were repeatedly mentioned by participants, and no new information emerged, data collection was concluded. Two stakeholders were suggested by others, but we were unable to schedule meetings with them due to their busy schedules.

For the interviews with medical doctors, the inclusion criteria were as follows: participants had to be currently working in the hospital, be responsible for the discharge process of patients, and be affiliated with the internal medicine department. There were no restrictions based on level of qualification; participants included house officers (interns), registrars (under specialty training), medical officers (not under training), and consultants/specialists. Additionally, there were no restrictions on the duration of their work in the department. Doctors working in the emergency department () department were excluded, as they are responsible for direct discharge from emergency and do not follow the same processes. For stakeholder participants, we initially recruited directors of curative medicine and directors of hospital management at both the federal and state levels. Further stakeholders were identified using a snowball sampling approach, based on recommendations made by medical doctor participants during interviews. A total of 17 participants were recruited, including 10 medical doctors, 3 state stakeholders, and 4 federal stakeholders Table 1.

**Table 1.** List of the participants.

Participants	Job position titles	Place of work
P1	Physician consultant	Ibrahim Malik Teaching Hospital
P2	Physician consultant	Ibrahim Malik Teaching Hospital
P3	Medical doctor (undertraining for internal medicine specialty)	Ibrahim Malik Teaching Hospital
P4	Medical doctor (undertraining for internal medicine specialty)	Ibrahim Malik Teaching Hospital
P5	Medical doctor (undertraining for internal medicine specialty)	Ibrahim Malik Teaching Hospital
P6	Medical doctor (undertraining for internal medicine specialty)	Ibrahim Malik Teaching Hospital
P7	House officer (in the internal medicine department)	Ibrahim Malik Teaching Hospital
P8	House officer (in the internal medicine department)	Ibrahim Malik Teaching Hospital
P9	House officer (in the internal medicine department)	Ibrahim Malik Teaching Hospital
P10	Family medicine specialist	Omar Ibn Al Khattab Primary Health Care Center
P11	Head of a directorate	Federal ministry of health (FMOH)
P12	Head of a directorate	FMOH
P13	Head of a directorate	FMOH
P14	Head of a directorate	FMOH
P15	Head of a directorate	State Ministry of Health
P16	Head of a directorate	State Ministry of Health
P17	Head of a directorate	State Ministry of Health

### Data Collection

The first author (A.M.) conducted semi-structured interviews (Supplemental S2 File: Interview guide). A semi-structured interview guide was used for data collection. The guide was developed using constructs from 3 key frameworks: the Model for Understanding Success in Quality (MUSIQ),^24^ the Care Transitions Framework (based on the Consolidated Framework for Implementation Research),^25^ and the WHO Framework on Integrated People-Centered Health Services (IPCHS).^26^ The interview guide was not pilot tested before data collection. The interviews were face-to-face interviews in the workplaces of the participants. Each interview lasted between 45 and 60 minutes. The interviews were conducted in Sudanese Arabic language and the interviewer and interviewees used English terminology when it was needed. All interviews were recorded and transcribed verbatim (S3 File: Table of participants’ quotations).

### Data Analysis

#### Identification of Underlying Challenges and Their Influencing Factors

We conducted thematic content analysis using a deductive coding approach to identify the challenges and the factors affecting follow-up care after hospital discharge. Quotations were coded if they described events or scenarios that furthered the understanding of how the contextual barriers and facilitators interact to improve follow-up care for chronic disease patients after hospital discharge. The analysis consisted of 3 steps: content line-by-line coding, development of categories from group codes, and development of the analytical themes (thematic analysis).^27^ We grouped the factors into 4 categories to organize the factors as they relate to the health system.

#### Development of the Causal Loop Diagram

The causal loop diagrams were developed based exclusively on the qualitative data from the interviews. First, we used purposive text analysis to develop CLDs. This approach involves systematically reviewing key informant transcripts, extracting quotations that describe drivers affecting follow-up care after hospital discharge, and extracting cause-and-effect statements, with diagrams that represent these relationships. Atlas.ti was used to manage data and Vensim (PLE) was used to produce CLDs. These variables were iteratively mapped using systems thinking principles to visualize feedback loops and dynamic interactions relevant to the discharge and follow-up care process. CLDs feature variables with measurable values, causal linkages (arrows showing variable interactions), and feedback loops that reinforce or balance changes.^28^ Then we validated and refined the first draft CLD in 3 meetings with 2 of the initial participants, 1 being a medical doctor, and the other being a stakeholder at the federal level, the validation period of the CLD started in July 2022 and ended in August 2023. The objective of the meeting was to examine the diagram variables and relationships and describe any missing variables or relationships. The participants received the causal loop drafts 2 weeks before the meeting. At each meeting, the last version of the diagram was presented. The participants discussed the phrasing and meaning of variables, and the nature of the relationships and identified missing variables and relationships. The diagram was then refined based on the feedback. The validation process was guided by the CLD validation tool.^29^

## Results

**Figure 1.**
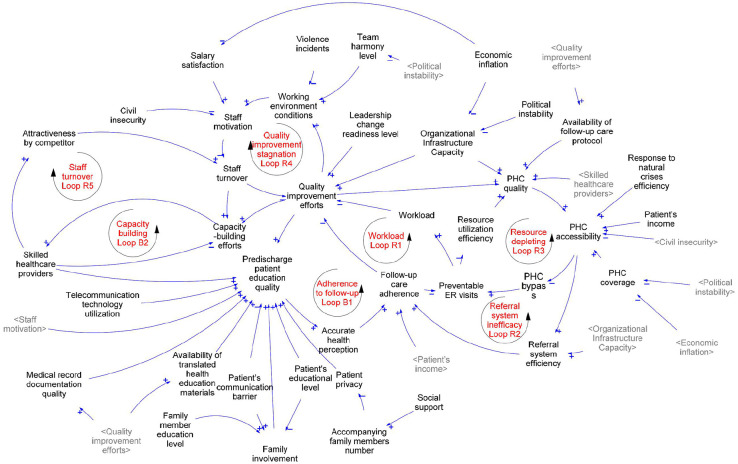
A causal loop diagram shows all the factors that influence adherence to follow-up care after hospital discharge.

The thematic analysis revealed 5 major themes that describe the underlying challenges affecting follow-up care after hospital discharge and 40 other interrelated factors from the perspectives of physicians and key healthcare stakeholders: follow-up care adherence, quality of patient education, referral system efficiency, primary healthcare (PHC) accessibility, and quality improvement efforts. We grouped these factors into 4 categories: 10 personal factors, 11 program factors, 13 health sector factors, and 4 intersectoral factors. Table 2 presents the descriptive definition of the factors and their relations to other factors. Figure 1 presents the CLD, which illustrates the factors that influence follow-up care after hospital discharge. We identified 5 reinforcement feedback loops (R) and 2 balancing loops (B) affecting follow-up care after hospital discharge. To support readers’ understanding of the diagram, factors have been underlined in the narrative description.

**Table 2.** Definition and links of the causal loop factors.

No	Factor	Factor classification	Description	Inflow variables^a^ (relationship type +/−)	Outflow variables^b^ (relationship type +/−)
1.	Follow-up care adherence	Program factors	Follow-up care adherence refers to the extent to which a patient complies with or adheres to the recommended schedule and instructions after hospital discharge. Follow-up care aims to: • Monitor the patient’s recovery. • Manage ongoing or emerging health issues. • Prevent emergency room (ER) visits and hospital readmissions. • Address potential complications at an early stage.	Patient’s income (+), Referral system efficiency (+), Accurate health perception (+)	Preventable ER visits (−), Quality improvement efforts (+)
2.	Pre-discharge patient education quality	Program factors	As part of the discharge process, medical doctors conduct health education sessions that include: • Explaining prescribed medications. • Providing instructions for necessary post-discharge care. • Discussing potential further investigations. • Scheduling follow-up visits at the outpatient clinic.	Family involvement (+), Patient’s communication ability (+), Quality improvement efforts (+), Availability of translated health education materials (+), Qualified healthcare providers (HCPs) (+), Patient’s education level (+), Staff motivation (+), Patient’s privacy (+), Medical record documentation quality (+), Telecommunication technology utilization (+)	Accurate health perception (+)
3.	Accurate health perception	Personal factors	Healthcare providers (HCPs) highlighted that: • Patients’ biased perception of their health can reduce their motivation to seek follow-up care, as they may believe they are already well. • Providing good quality counseling and education can help improve patients’ understanding of their actual health status and the need for continued care.	Pre-discharge patient education quality (+)	Follow-up care attendance (+)
4.	Quality improvement efforts	Health sector factors		Follow-up care adherence (+), Leadership change readiness level (−), Organizational infrastructure capacity (−), Staff turnover (−), Workload (−)	Pre-discharge patient education quality (+), Availability of translated health education materials (+), Working environment conditions (+), Medical record documentation quality (+), PHC quality (+), Capacity-building efforts (+), Availability of follow-up care protocol (+)
5.	Organizational infrastructure capacity	Health sector factors	Organizational infrastructure capacity refers to the foundational elements necessary for efficient healthcare operations, including: • Availability of essential hospital equipment (e.g., emergency drugs and emergency equipment). • Access to ambulances and established call centers for referral systems. • Maintenance of hospital hygiene standards and comfortable restroom facilities. • A robust documentation and archive system to store vital data (e.g., meeting minutes, written policies). • Effective governance through comprehensive policies to coordinate interactions between federal, state, and local entities.	Economic inflation (−), Political instability (−),	Referral system efficiency (+), PHC quality (+), Quality improvement efforts (+)
6.	Leaders change readiness	Personal factors	Resistance to change refers to a personal or organizational reluctance to adapt to new circumstances or practices, characterized by: • Individuals or groups being unwilling to change established ways of working. • Top stakeholders’ mindsets and resistance acting as major barriers to sustaining improvement efforts within the health system. • The need for effective communication and leveraging personal networks to promote understanding and acceptance of change.		Quality improvement efforts (+)
7.	Skilled HCPs	Health sector factors		Capacity-building efforts (+)	Attractiveness by competitor (+), PHC quality (+), Capacity-building efforts (−), Pre-discharge patient education quality (+)
8.	Staff turnover	Health sector factors	Staff turnover occurs due to factors such as: • Migration of healthcare workers. • Reassignment of employees to other health facilities offering better working conditions or higher motivation.	Attractiveness by competitor (+), Staff motivation (−)	Capacity-building efforts (+), Quality improvement efforts (−)
9.	Staff motivation	Personal factors	• Feeling emotionally and physically drained, leading to reduced engagement in improvement activities over time. • That poor working conditions in hospitals negatively affect their motivation and willingness to participate in system improvement efforts.	Salary satisfaction (+), Civil insecurity (−) Working environment conditions (+)	Staff turnover (−), Pre-discharge patient education quality (−)
10.	Capacity-building efforts	Health sector factors	Training initiatives to improve patient care are typically: • Organized by the Continuous Professional Development (CPD) department at the Federal Ministry of Health (FMOH). • OR conducted by CPD departments within hospitals.	Staff turnover (+), Quality improvement efforts (+), Skilled HCPs (−)	HCPs (+)
11.	Work environment conditions	Health sector factors	Working conditions encompass factors such as: • Work schedules. • Organization of job tasks. • Availability of training and professional development opportunities. • Presence of health and safety hazards.	Quality improvement efforts (+), Violence incidents (−), Team harmony level (+)	Staff motivation (+)
12.	Team harmony level	Program factors	Team harmony level refers to the degree of cohesion and cooperation among team members, influenced by: • Political instability, which can create divisions among staff due to differing political affiliations. • Increased staff turnover when individuals feel misaligned or in opposition to dominant political groups. • Some providers relying on personal or professional connections to access resources, fulfill responsibilities, and overcome challenges in politically unstable environments.	Political instability (−)	Work environment conditions (+)
13.	Violence incidents against HCPs	Health sector factors	The HCPs states that the possibility of being exposed to violence from patients and their families affects their motivation to work		Work environment conditions (−)
14.	Salary satisfaction	Health sector factors		Staff motivation (+)	Economic inflation (−)
15.	Workload	Program factors	When the workload is high the HCPs do not have time to focus on improving the quality.	Quality improvement efforts (−)	Preventable ER visits (+)
16.	Telecommunication technology utilization	Program factors	Healthcare providers highlighted the role of telecommunication technology in facilitating follow-up care, including: • Use of WhatsApp and phone calls to enable remote communication between doctors and patients. • Voluntary efforts by medical doctors to contact patients who are illiterate or at high risk of complications and require close monitoring after discharge.		Pre-discharge patient education quality (+)
17.	Medical record documentation quality	Program factors	Healthcare providers described documentation practices for follow-up care as: • Hospitals using paper-based discharge summaries, typically written in English, to communicate patient information between doctors. • Primary healthcare (PHC) facilities lacking formal recording systems and relying on notebooks for documenting baseline readings and follow-up notes. • Patients being responsible for purchasing and maintaining these notebooks.	Quality improvement efforts (+)	Pre-discharge patient education quality (+)
18.	Referral system efficiency	Health sector factors	The referral system is designed to guide patient care transitions, where: • The patient’s first contact is with primary healthcare (PHC) facilities. • Healthcare providers use a formal referral process, starting with Form A, which transfers the patient from PHC to the hospital. • Upon discharge, patients receive Form B, serving as feedback from the hospital to the PHC. • If referred to a tertiary hospital, Form B is again used to transfer the patient back to the PHC after hospital care.	PHC accessibility (+), Organizational infrastructure capacity (+)	Follow-up care adherence (+)
19.	PHC quality	Program factors	The availability of quality basic lab tests, skilled HCPs, basic affordable medication, and water supply and electricity	Quality improvement efforts (+), Availability of follow-up care protocol (+), Organizational infrastructure capacity (+), Availability of follow-up care protocol (+), Skilled HCPs (+), Resource utilization efficiency (+)	PHC accessibility (+)
20.	Availability of follow-up care protocol	Program factors	At the PHC the HCPs might struggle to find standardized care protocols availability of these protocols would facilitate the follow-up carte in PHC	Quality improvement efforts (−)	PHC quality (+)
21.	PHC bypass	Health sectors factors	When the patients skip the PHC as the first point of contact and they go directly to the hospital	PHC accessibility (−)	Preventable ER visits (+)
22.	PHC accessibility	Health sector factors	Accessibility to PHC is affected by the quality of PHC and geographical coverage of PHC service	Response to natural crises efficiency (+), Civil insecurity (−), PHC coverage (+), PHC quality (+), Patient’s income (+)	Referral system efficiency (+), Bypass the PHC (−)
23.	PHC coverage	Health sector factors	One of the factors that influence the accessibility to the PHC is geographical coverage. There are several projects aimed at addressing the service coverage problem of the health system through an accelerated strengthening of services either through an increasing number of PHCs or an increasing package provided within the PHC.	Civil insecurity (−), Political instability (−)	PHC accessibility (+)
24.	Preventable ER visits	Health sector factors	These are incidents that HCPs aim to minimize	PHC bypass (+), Follow-up care adherence (−)	Resource utilization efficiency (−), Workload (+)
25.	Resource utilization efficiency	Health sector factors	Factors influencing accessibility to PHC include geographical coverage, with efforts to address this through: • Increasing the number of primary healthcare centers (PHCs). • Expanding the range of services provided within existing PHCs through targeted health system strengthening projects.	Preventable ER visits (−)	PHC quality (+)
26.	Family involvement	Program factors	From the perspective of HCPs, family involvement is associated with patients’ ability to communicate effectively and the necessity for their families to provide financial support.	Family member education level (+), Homeless patients (−), Patient’s communication ability (+), Patient’s education level (−), Patient’s income (−)	Pre-discharge patient education quality (+)
27.	Patient’s privacy	Program factors	Ensuring privacy during the counseling sessions is crucial for delivering high-quality patient care and it facilitates patient doctors’ communication	Accompanying family members’ number (−)	Pre-discharge patient education quality (+)
28.	Accompanying family members’ number	Personal factors	Due to the huge responsibility on the family during the admission to like to bring lab results or medication from the medicine, this brings the tradition that a lot of patients accompanying the patients to do those tasks	Social support level (+)	Patient’s privacy (−), Family involvement (−)
29.	Social support level	Personal factors	The HCPs perceive that the number of visitors and family involvement is influenced by cultural social support norms. This social support not just visiting the patients it also included emotional and financial responsibility toward the patients and their close family		Accompanying family members’ number (+)
30.	Patient’s education level	Personal factors			Family involvement (−), Patient counseling quality (+)
31.	Family member education level	Personal factors			Family involvement (+), Pre-discharge patient education quality (+)
32.	Availability of translated health education materials	Program factors	The absence and inaccessibility of educational materials in the patient’s native language (Arabic), can indeed pose a significant barrier to effective healthcare communication and self-management.	Quality improvement efforts (+)	Pre-discharge patient education quality (+)
33.	Patient’s income	Personal factors	Healthcare providers (HCPs) noted that financial constraints impact continuity of care by: • Limiting patients’ ability to afford transportation for follow-up visits. • Reducing access to prescribed medications. • Hindering the ability to undergo further necessary medical investigations after hospital discharge.		Follow-up care adherence (+), PHC accessibility (+)
34.	Homeless patients	Personal factors	Those who live mostly on the street		Family involvement (−)
35.	Patient’s communication ability	Personal factors	Communication barriers due to health conditions like being elderly, or having dementia, deafness, etc		Family involvement (+), Pre-discharge patient education quality (−)
36.	Economic inflation	Intersectoral factors			Organizational infrastructure capacity (−), Salary satisfaction (−), PHC coverage (−)
37.	Civil insecurity	Intersectoral factors	Insecurity is usually due to conflict or war. Insecurity is affecting access to PHC, and the safety of patients and HCPs becomes a primary concern. It also leads to turnover of the HCPs when the conflict extends to the main big cities of Khartoum and Madani		Staff motivation (−), PHC accessibility (+)
38.	Political instability	Intersectoral factors	Political instability, as defined in this study, includes: • Unforeseen or unexpected events such as the end of a government or an electorate, whether legally or by force. • Chronic protest movements resulting in the system’s prolonged inability to perform basic functions. • Manifestations such as continuous protests or doctor strikes, which hinder physical access to follow-up healthcare services.		Organizational infrastructure capacity (−), Team harmony level (−), PHC coverage (−)
39.	Response to natural crises efficiency	Intersectoral factors	Natural crisis: torrent, heavy rainy seasons, and floods can isolate villages and small cities and can limit accessibility to healthcare services.		PHC accessibility (+)

a Factors that affect the theme. The direction of the relationship is inflow.

b Factors that the theme affects. The direction of the relationship is outflow.

### The Underlying Challenges

#### Follow-Up Care Adherence

Following hospital discharge, medical doctors usually schedule a follow-up visit at the outpatient clinic within the first 2 weeks post-discharge. However, adherence to this recommended schedule is often lacking, which increases the possibility of ER visits. This in turn increases workload and hinders quality improvement efforts. Patients’ accurate perception of their health and understanding of the diagnosis can influence their decision to attend follow-up visits. Low adherence to follow-up care necessitates quality improvement efforts to improve the pre-discharge education of patients. The income level of the patients affects their motivation to seek further care; financial constraints, such as limited resources for transportation or treatment can hinder their adherence to follow-up care. The ineffectiveness of current referral systems has resulted in inadequate communication between healthcare providers (HCPs) and thus led to uncoordinated care between PHCs and hospitals, thereby diminishing the likelihood of follow-up care.

#### Pre-Discharge Patient’s Education Quality

As part of the discharge process, doctors provide pre-discharge education sessions. These sessions include explanations of medications, necessary post-discharge care, possible further investigations, and scheduling of outpatient follow-up visits. The quality of health education depends on the skills of the doctors, and whether they are registrars or house officers, who have direct contact with the patient. Working conditions such as long working hours, time constraints and work overload can have a significant impact on the motivation of doctors to deliver these sessions to a high standard. The availability of essential hospital equipment, adequate hospital hygiene, and doctors’ break rooms are among the infrastructural factors that improve motivation. The absence of translated educational materials in Arabic reduces the quality of the session. Ensuring patient privacy during counseling sessions is fundamental to providing quality education. This can be compromised in facilities that do not have adequate space for private counseling. The presence of many visitors, who could provide social support for patients, can reduce privacy, leaving the HCPs without the space to provide information and discuss sensitive patient concerns. Low levels of education, or patients with communication barriers (eg, elderly, dementia, deafness) may reduce the quality of the meeting, while these circumstances increase the likelihood of family involvement. The use of telecommunication technology (using telephone calls and WhatsApp messages) emerges as an important factor in facilitating patient and family education.

#### Referral System Efficiency

The patient pathway is typically defined by a formal referral system, where the patient enters the hospital referred from the PHC of their catchment area. HCPs use paper-based communication (Form A and Form B). Form A includes information about the patient’s medical history and the reason for referral, and it is used to communicate patient information from the PHC to the hospital. After hospital discharge, patients should be referred back to the PHC using another similar paper-based communication, Form B, which outlines the patient’s course in the hospital. However, in current practice, patients directly approach the hospital without being referred from the PHC and bypass the PHC. HCPs in the hospital don’t typically send patients back to the PHC, as PHCs are often inaccessible to the patients, and patients are kept seen in the outpatient clinic for a limited time. Subsequently, patients with chronic diseases may present to the ER with complications. The referral system’s efficiency is hindered by several organizational infrastructural barriers, including weak governance, inadequate accountability systems, and substandard infrastructure; there is insufficient availability of ambulances and essential healthcare equipment, such as oxygen supplies and beds. Constant economic inflation has also affected infrastructure and facility improvements.

#### PHC Accessibility

Accessibility to PHC is highlighted as one of the challenges in ensuring follow-up care after hospital discharge. Economic inflation and political instability undermine PHC coverage. The lack of governance and accountability influences the construction of new PHCs, often driven by the personal interests of politicians in power at the time. These influences may lead to prioritizing building hospitals over PHCs, changing the planned site for a PHC, or even neglecting the construction of PHCs altogether. Even when PHCs are available, they may offer services of low quality due to various factors, including a high turnover of skilled HCPs, the absence of guidelines or clinical protocols for post-hospital discharge follow-up care, or insufficient quality improvement efforts. Patients’ access to PHC services is further hampered by natural disasters such as floods and mudslides, civil insecurity due to armed conflicts or continuous protest movements, or financial constraints.

#### Quality Improvement Efforts

Improving the quality of pre-discharge patient education in PHC and improving the working environment conditions require dedicated quality improvement efforts. However, such initiatives face significant challenges. In the context of political instability, leaders face job insecurity which diminishes team harmony, thereby affecting the work environment condition and contributing to decreased motivation and higher turnover rates. Stakeholders have indicated that disruptions in team harmony force them to rely on personal networks for support. Inadequate hospital infrastructure, long working hours, and violence against staff negatively affect the working environment and motivation of the HCPs, ultimately leading to high staff turnover. Staff turnover has increased recently due to civil unrest, low salaries, and economic inflation. There is a continuous need for capacity-building efforts due to the leakage of skilled HCP turnover. Quality improvement efforts may struggle to be sustained due to the change-resistant mindset of some stakeholders and a lack of effective leadership. Moreover, quality improvement efforts require sufficient allocation of resources and robust organizational infrastructure, including, effective information management systems, governance, and accountability mechanisms.

### Detail of Feedback Loops

**Figure 2.**
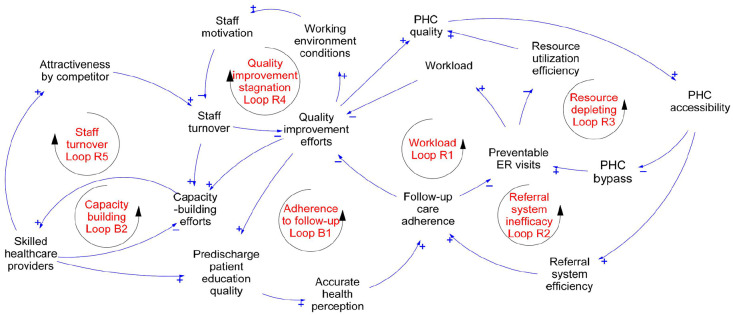
A causal loop diagram with a focus on the reinforcing and balancing loops.

We identified 2 balancing loops and 5 reinforcing loops (Figure 2). The first balancing loop is **adherence to follow-up care balancing loop B1**. The goal is high adherence, which is not achieved. This gap necessitates investment efforts to improve the quality of patient care, including enhancing the quality of patient education. Having patients with realistic perceptions of their health would eventually improve adherence to follow-up care. However, we identified reinforcing loops that would counteract these efforts. The initial low quality of pre-discharge patient education contributes to a biased perception of health, ultimately reducing adherence to follow-up care. This lack of adherence leads to an increase in preventable ER visits, consequently increasing the providers’ workload and lowering the possibility of quality improvement efforts **(workload reinforcing loop R1**). Additionally, the inefficient referral system reduces adherence to follow-up care, thereby increasing the occurrence of later ER visits and depleting the resources for enhancing the quality of PHC, reducing access to PHC services, and perpetuating an inefficient referral system **(referral system inefficiency reinforcing loop R2).** Another loop occurs when patients bypass primary healthcare facilities in favor of direct visits to the ER for services that could be provided at the PHC level. This bypass is often attributed to the low quality of PHC services, resulting in limited access to PHC facilities. Despite services being more cost-effective at the PHC level, patients seek care at the ER, burdening the healthcare system. Consequently, essential tasks and services that could be efficiently managed at the primary healthcare level are needlessly shifted to hospitals, leading to depleted resources and compromised service quality at both PHC facilities and hospitals **(resource-depleting reinforcing loop R3).** When quality improvement efforts stagnate, staff lose motivation, leading to increased staff turnover. The more staff turnover, the more quality improvement efforts stagnate or fail **(quality improvement stagnation reinforcing loop R4)**. The staff turnover reinforcing loop undermines the capacity-building efforts balancing loop. Capacity building increases the number of skilled HCPs; if the number of skilled HCPs increases, the need for capacity building decreases **(capacity building balancing loop B2)**. While these initiatives aim to produce skilled HCPs, the reality of these efforts also leads to the attraction of competing employers (inside or outside the country), leading the staff turnover. Therefore, staff turnover necessitates ongoing capacity-building efforts **(staff turnover reinforcing loop R5)**.

## Discussion

### Results Interpretation

Our study engaged a diverse group of healthcare professionals, including physician consultants, doctors in training, house officers, and senior health administrators. These participants were drawn from a major urban teaching hospital, a primary healthcare center, and both federal and state Ministries of Health. Their experience ranged from early-career clinicians to senior policymakers, offering a multifaceted view of the discharge and follow-up care process. Physicians at the hospital emphasized clinical workload, limited patient understanding, and documentation practices, while stakeholders from the Ministries highlighted systemic challenges such as weak referral mechanisms, high staff turnover, and governance limitations. In this study, we used the CLD to examine the factors affecting the continuity of follow-up care in patients with chronic conditions after hospital discharge in Sudan. We found that continuity of follow-up care is challenged by low adherence. Notably, ensuring the continuation of follow-up care requires high-quality education during the hospital stay and at discharge, effective referral systems between the hospital and PHC, accessibility of the PHC, and continuous quality improvement efforts. Quality improvement efforts can offset the challenge of low adherence to follow-up care. The identified reinforcing feedback loops impede progress toward quality improvement solutions.

A scoping review of transition-of-care interventions found that self-management support was a consistent component in all effective interventions aimed at improving transitional care.^30^ This finding was echoed in another study involving patients with chronic disease conducted in Sudan, where one of the main needs identified by patients during the transition from hospital to home was to feel well informed.^22^ However, meeting this need remains a significant challenge. Our research found that the quality of predischarge education is low and there are several barriers to providing adequate self-management support.

Follow-up sessions are an important part of the management of patients with chronic conditions and allow patients to be educated and strengthen their self-management skills. Evidence suggesting that the quality of pre-discharge education sessions and the use of telecommunication can enhance patients’ self-management skills.^31,32^ For patients with chronic diseases, care extends beyond hospital discharge, with follow-up care being integral to the admission process, particularly within 30 days post-discharge.^33^ Neglecting follow-up care during this critical period can result in complications and emergency readmissions.^34^ Follow-up visits with hospital physicians and later in the nearest PHC play a pivotal role in mitigating the adverse outcomes during the 30-day post-discharge period.^35,36^ The level of self-awareness and proficiency in self-management skills correlates with successful follow-up care.^37^ Structured educational sessions provided during hospital discharge have been shown to enhance patients’ self-management abilities, especially beneficial for those with chronic conditions.^38,39^ Conversely, the absence of such support leaves patients ill-equipped to manage their health challenges effectively.^40^

The study emphasizes how the quality of the PHC significantly impacts access to the PHC. Insufficient availability or low-quality services often drive patients, especially those with chronic illnesses, to seek care in hospitals, exacerbating the strain on resources. This reliance on hospital facilities perpetuates a cycle of resource depletion, underscoring the urgent need to improve PHC quality (Feedback loops 1 and 2). Research shows that improving healthcare service quality leads to greater reductions in mortality rates than merely expanding service coverage, highlighting the importance of prioritizing quality improvement initiatives in healthcare delivery systems.^41^ The HCPs reported using telecommunication (WhatsApp calls) to communicate with specific patients, considering it a facilitator for information exchange and follow-up visits. This communication, however, typically lasts for a maximum of 3 to 4 months. House officers and medical residents, who typically spend 3 to 6 months in the department, are the most likely to share their phone numbers. A study has shown that completing the first 2 calls after hospital discharge was associated with increased patient compliance with follow-up activities, suggesting that making 2 to 3 phone calls to patients is optimal for effective care transition.^42^

Factors like economic inflation and civil unrest significantly impact staff turnover and service delivery, exacerbating existing issues. Addressing these challenges requires coordinated action across sectors. Instances of violence targeting medical professionals worsen staff shortages in already strained facilities.^43^ Implementing multisectoral policies to retain trained staff is crucial to mitigate these challenges.^44-46^

Our findings underscore the link between HCPs’ motivation and quality improvement efforts, highlighting how organizational challenges can hinder both. Studies show that while motivated staff are essential for guaranteeing the quality of medical care (41, 43), quality improvement programs might have a limited impact on motivation.^47^ As health policymakers, donors, and private institutions work to improve the quality of care, they must recognize the importance of engaging healthcare workers in the design, development, and implementation efforts to deliver better outcomes and sustainability of interventions.^48^ Involving healthcare workers is also a way to prevent staff burnout, according to previous research at the same hospital.^49^

A modified Delphi study involving international experts highlighted the critical role of leadership engagement and stakeholder involvement in the successful implementation of transitional care innovations.^50^ The administrative leaders reported that they are affected by job instability due to political changes. Consequently, they tend to prioritize short-term strategies, potentially neglecting crucial developmental initiatives due to the fear of losing their positions before these longer-term projects come to fruition. Studies indicate that political instability negatively impacts job performance and can result in the failure of quality improvement programs.^51-53^ Political commitment is crucial for quality improvement in healthcare in low- and middle-income countries.^54^ Successful quality improvement requires a shared vision, reliable data, and an organizational culture of accountability. Political commitment is essential in addressing these challenges, as it sets the tone for accountability.^55^ A study highlighted that political instability leads to frequent rotation and turnover of staff, lack of handover procedures, and impact on the quality and quantity of human resources for health in persons working on HIV/AIDS in Guinea-Bissau.^56^ The stakeholders reported that resistance during quality improvement project implementation often prompted them to seek support within their personal networks. While this approach may temporarily resolve issues, it can inadvertently exclude opposing voices, hindering collaboration, and sustainable project success. Recent research suggests that resistance to change can be overcome by addressing key stakeholders’ cognitive beliefs and positive emotions about change.^57^

### Practical Implications

The understanding of how factors related to continuity of follow-up care after hospital discharge are interconnected, and their combined impact on follow-up care complexity, can be used to design effective interventions. Key practical recommendations focus on addressing crucial leverage points in the system. The study suggests strengthening the organizational challenges to address staff motivation and turnover. We recommend combining continuous professional development efforts with efforts to create a supportive work environment. The study also suggests enhancing the quality of PHC by establishing and evaluating chronic disease management guidelines, defining essential care components, and ensuring affordability. We recommend standardizing and incentivizing telecommunication use, investing in technology infrastructure and training to improve post-discharge coordination. Finally, the study suggests leveraging digital technologies to improve referral systems, which can enhance access to PHC and follow-up care, with further research needed on the impact of digital health interventions.

### Strengths and Limitations

Strengths of this approach include the representation of key stakeholders and the synthesis of a complex problem into a visual causal map. There may be inherent limitations to this method. While focusing on a specific region adds depth, it also raises concerns regarding generalizability. The findings, grounded in Khartoum State, may not be fully generalizable to other regions of Sudan. This study explored follow-up care for chronic diseases in general, without focusing on any specific condition. This should be considered when interpreting the findings. Variations in healthcare infrastructure, cultural practices, and socioeconomic conditions across different areas may influence the continuity of follow-up care in distinct ways. Due to limited resources, we did not employ an interactive workshop for group model building, which could allow for interactive building between participants. To mitigate this limitation, we asked participants to validate the first version of the causal loop. Although we included various categories of doctors and administrative stakeholders, the perspective of allied healthcare providers, such as nursing staff, is missing. In addition, we acknowledge that this study does not include the perspectives of patients or caregivers, which are critical to capturing the full scope of challenges and needs in the follow-up care process after hospital discharge.

### Implications for Future Research

Future studies could benefit from a mixed-methods approach that integrates both qualitative insights and quantitative data to provide a more comprehensive understanding of follow-up care challenges. This could include data on hospital readmission rates, access to outpatient services, medication adherence, and healthcare worker availability. Such integration would help quantify the burden of poor transitional care and identify system-level gaps more precisely. Future longitudinal studies that track patients and system interactions over time could help uncover the evolving challenges and facilitators of follow-up care, providing a more dynamic understanding to inform sustainable health system improvements. Future studies are also needed to explore these contextual differences and assess the applicability of our findings in other regions.

## Conclusion

In this study, we utilized a causal loop diagram to illustrate the factors influencing follow-up care for patients with chronic diseases after hospital discharge in Sudan, revealing that low adherence to follow-up care is a major issue. There’s a focus on training HCPs without addressing underlying issues. Redirecting efforts toward improving the quality of pre-discharge patient education could be beneficial, however, the workload, resource-depleting, referral system inefficacy, and quality improvement stagnation reinforcing loops hinder progress in this direction. Addressing these challenges requires multifaceted approaches, including enhancing the quality of the PHC, leveraging digital technology for referral system improvement, and tackling organizational challenges affecting staff motivation and turnover. Overall, our findings provide a foundation for evidence-informed policies and interventions aimed at reinforcing follow-up of care after hospital discharge in Sudan.

## Supplemental Material

sj-docx-1-his-10.1177_11786329251349916 – Supplemental material for Contextual Factors Affecting Continuity of Follow-Up Care After Hospital Discharge for Patients with Chronic Diseases in Sudan: A Qualitative Study with Causal Loop Diagram Insights Supplemental material, sj-docx-1-his-10.1177_11786329251349916 for Contextual Factors Affecting Continuity of Follow-Up Care After Hospital Discharge for Patients with Chronic Diseases in Sudan: A Qualitative Study with Causal Loop Diagram Insights by Asma MohamedSharif and Armin Gemperli in Health Services Insights

sj-docx-2-his-10.1177_11786329251349916 – Supplemental material for Contextual Factors Affecting Continuity of Follow-Up Care After Hospital Discharge for Patients with Chronic Diseases in Sudan: A Qualitative Study with Causal Loop Diagram Insights Supplemental material, sj-docx-2-his-10.1177_11786329251349916 for Contextual Factors Affecting Continuity of Follow-Up Care After Hospital Discharge for Patients with Chronic Diseases in Sudan: A Qualitative Study with Causal Loop Diagram Insights by Asma MohamedSharif and Armin Gemperli in Health Services Insights

sj-docx-3-his-10.1177_11786329251349916 – Supplemental material for Contextual Factors Affecting Continuity of Follow-Up Care After Hospital Discharge for Patients with Chronic Diseases in Sudan: A Qualitative Study with Causal Loop Diagram Insights Supplemental material, sj-docx-3-his-10.1177_11786329251349916 for Contextual Factors Affecting Continuity of Follow-Up Care After Hospital Discharge for Patients with Chronic Diseases in Sudan: A Qualitative Study with Causal Loop Diagram Insights by Asma MohamedSharif and Armin Gemperli in Health Services Insights
