# Supplementary material for: Contextual Factors Affecting Continuity of Follow-Up Care After Hospital Discharge for Patients with Chronic Diseases in Sudan: A Qualitative Study with Causal Loop Diagram Insights
Source: Health Serv Insights. 2025 Jun 24;18:11786329251349916. doi: 10.1177/11786329251349916 (PMC12188063; doi:10.1177/11786329251349916)
Supplement: sj-docx-2-his-10.1177_11786329251349916 – Supplemental material for Contextual Factors Affecting Continuity of Follow-Up Care After Hospital Discharge for Patients with Chronic Diseases in Sudan: A Qualitative Study with Causal Loop Diagram Insights [file sj-docx-2-his-10.1177_11786329251349916.docx]

**Research project**

**Study continuity of care for chronic disease patients after hospital discharge and associated contextual factors in Sudan.**

INDIVIDUAL INTERVIEWS

**Micro level**

## Topic guide for key stakeholder interviews:

Research objectives for medical doctors’ interviews:

The following guide does not contain all the pre-set questions but rather lists the key themes and sub-themes to be explored with each participant. This allows the interviewer to formulate questions which are responsive to each individual participant. The topic guide does not include all follow-up questions like ‘why’, ‘when’, ‘how’, etc. as it is assumed that participants’ contributions will be fully explored throughout in order to understand how and why views, behaviors and experiences have arisen. While all topics will be covered with each participant, the order in which issues are addressed and the amount of time spent on different themes will vary between participants.

## The aim of the interview is to understand the contextual factors which influence the current practice and how they can facilitate interventions

1. Description of the discharge processes
2. Perceptions of about the continuity and integration of care after discharge
3. Facilitators and barriers in the current process of patients discharge
4. Examples of recent successful and unsuccessful discharge
5. Perceived facilitators, barriers and opportunities for future improvement
   - Introduce self.
   - Introduce research (funding, research design, outputs).
   - Explain: confidentiality, tape recording, length of interview, nature of discussion (specific topics to address, but conversational in style, in your own words, no right or wrong answers), reporting and data storage/archiving.
   - Any questions.
   - Obtain written consent.

| **Aim** | **Main Questions** | **Probes** | **Interviewer notes** |
| --- | --- | --- | --- |
| **Introduction & warmup** | To start off, I’d love to learn a little bit about  your background.  Where were you before [current hospital]? What do you do at [hospital]? |  |  |
| **Description of the discharge processes** | Let’s start by having you briefly describe what you do at the hospital.  Please describe your role and experiences with patient pre, at and post discharge  How you engage the patient and the family? | What is the process? Can you walk me through the process?  What is your role in this process? How does it get decided patients discharge? | **Are those interventions included?** Patient involvement Health education- patients empowerment. Medication management Discharge arrangements Coordination with other providers  Providing information and guidance to patient/family- Shared clinical decision making between individual, families and providers  Providing psychological and social support  Anticipation and preparation for emergencies/deterioration Feeling of safety |
| **Perceptions of about the continuity and integration of care after discharge.** | Tell me about the process of navigating the discharge patient from hospital to home. | Are there patient characteristics that play into the decision (target group)?  Could you give an example of discharge activities? | **How I look to the questions?**   1. Identify two to three problems    - for the whole activity system    - between subsystems |

|  |  | How you ensure engagement of the patient and families?  Tell me about how you ensure integration of your care for complex patients?  How you ensure continuity of care after discharge?  How long does the process of? discharging a patient take?  What is expected of you during discharge? | - within each the sub-systems   1. Identify 2-3 co-factors for each problem. 2. Determine the stakeholder system that is responsible for each co- factor. |
| --- | --- | --- | --- |
| **Facilitators and barriers in the process of patients discharge Examples of recent successful and**  **unsuccessful discharge.** | What aspects of patient discharge work well? | What types of discharge work well? Why? Who/What has contributed to that success?  Can you give an example of a recent successful discharge?  What makes a transfer successful? |  |
|  | What aspects of patient transfers don’t  work so well? | What types of problem? Why? What are the particular barriers to these patients? Can you give an example of a recent problematic discharge?  What makes it problematic?  Have you or any other team members addressed these concerns? How did you go about it? Who were the key players? If you have not addressed the issue, ideally, how would you go about fixing it? | Factors from the literatures, I am looking for?  Leadership engagement Engaging key stakeholders Information continuity  Financing of TCIs’ implementation Available resources: hospital infrastructure, human resource management, Team for quality improvement  HIT systems  Access to knowledge and information: Training, counseling, and education |

Engaging organizations like medical societies, external context: Donor pressure

Policy pressure

e.g Affordable Care Act; nonpayment for readmissions in US

Sense of urgency:

Relative priority:

Reflecting and evaluating:

Role of incentive and payment system.

Norms, values, and basic assumptions of given unit(s) or organization(s) that affect views of the intervention and its implementation

Knowledge and beliefs - emphasize teamwork, communication,

freedom to make decisions, commitment to improve

Triggers = revolution and protesting Presence of a specific event (positive or negative) that stimulates a new

|  |  |  | emphasis on improving quality in the area of focus of a given QI project.  Task strategic importance to the organization. |
| --- | --- | --- | --- |
| **Opportunities for improvement and plans** | What should be done to improve the transition of the patients from the hospital to home? How to do? | Do you see opportunities for improvement in the current process of care? If so, could you share them?  How you think these interventions can be implemented? | **Proposed interventions:**  Home visit Telephone care  Personal care assessment and treatment plans  Medication reconciliation – add the pharmacist |
| **Unicorn wishes & wind down** | Is there anything else you could share with me that might help me better understand these transitions of care?  What should I have asked about?  Is there anyone else you think I should interview for this project?  Do you have any questions for me? |  |  |

**Research project**

**Study continuity of care for chronic disease patients after hospital discharge and associated contextual factors in Sudan.**

INDIVIDUAL INTERVIEWS

**Meso - Micro level**

# Topic guide for key stakeholder interviews:

Research objectives for medical doctors’ interviews:

The following guide does not contain all the pre-set questions but rather lists the key themes and sub-themes to be explored with each participant. This allows the interviewer to formulate questions which are responsive to each individual participant. The topic guide does not include all follow-up questions like ‘why’, ‘when’, ‘how’, etc. as it is assumed that participants’ contributions will be fully explored throughout in order to understand how and why views, behaviors and experiences have arisen. While all topics will be covered with each participant, the order in which issues are addressed and the amount of time spent on different themes will vary between participants.

# The aim of the interview is to understand the contextual factors which influence the current practice and how they can facilitate interventions

1. Description of the interventions, experience and best practices to reach continuity of care and integration of care after patients discharge
2. Facilitators and barriers in the current strategies
3. Examples of recent successful and unsuccessful strategies and interventions
4. Perceived facilitators, barriers and opportunities for future improvement
   - Introduce self.
   - Introduce research (funding, research design, outputs).
   - Explain: confidentiality, tape recording, length of interview, nature of discussion (specific topics to address, but conversational in style, in your own words, no right or wrong answers), reporting and data storage/archiving.
   - Any questions.
   - Obtain written consent.

| **Aim** | **Main Questions** | **Probes** | **Interviewer notes** |
| --- | --- | --- | --- |
| **Introduction & warmup** |  | To start off, I’d love to learn a little  bit about your background.  Can you tell me about your position and your responsibilities? |  |
| **Perceptions of the current interventions, experience and best practices for the continuity and integration of care after discharge.** | Let’s start by having you briefly describe what you do to ensure integration and continuity of care. Please describe your role and experiences with programs and strategies?  What are the strategies to engage the patient and the family? | 1. Identify two to three problems    - for the whole activity system    - between subsystems    - within each the sub-systems 2. Identify 2-3 co-factors for each problem. 3. Determine the stakeholder system that is responsible for each co-factor. | **Are those interventions included?**  Patient involvement Health education Patient empowerment  Medication management Discharge arrangements Coordination with other providers  Providing information and guidance to patient/family Shared clinical decision making between individual, families and providers Providing psychological and  social support |

|  |  |  | Anticipation and preparation  for emergencies/deterioration Feeling of safety |
| --- | --- | --- | --- |
| **Facilitators and barriers in the current strategies Examples of recent successful and unsuccessful strategies.** | What aspects of health service integration and continuation work well? | What types of integration work well? Why? Who/What has contributed to that success?  Can you give an example of a recent successful strategy?  What makes it successful? Or not |  |
|  | What aspects don’t work so well? | What types of problem? Why? What are the particular barriers to these patients?  Can you give an example of a recent problematic discharge?  What makes it problematic?  Have you or any other team members addressed these concerns? How did you go about it? Who were the key players?  If you have not addressed the issue, ideally, how would you go about fixing it? | Factors from the literature, I am looking for?  Leadership engagement Engaging key stakeholders Information continuity Financing of TCIs’ implementation  Available resources: hospital infrastructure, human resource management, Team for quality improvement |
|  |  |  | HIT systems  Access to knowledge and information: Training, counseling, and education |
|  |  |  | Engaging organizations like medical societies, external context: Donor pressure |

Policy pressure

e.g Affordable Care Act; nonpayment for readmissions in US

Sense of urgency: Relative priority: Reflecting and evaluating:

Role of incentive and payment system

Building strong primary care (PC)-based systems

Norms, values, and basic assumptions of given unit(s) or organization(s) that affect views of the intervention and its implementation

Knowledge and beliefs - emphasize teamwork, communication,

freedom to make decisions, commitment to improve

Triggers = revolution and protesting Presence of a

|  |  |  | specific event (positive or negative) that stimulates a new emphasis on improving quality in the area of focus of a given QI project.  Task strategic importance to  the organization. |
| --- | --- | --- | --- |
| **Opportunities for improvement** | What should be done to improve the transition of the patients from the hospital to home? How to do? | Do you see opportunities for improvement in the current process of care? If so, could you share them? How you think these interventions can be implemented? | **Proposed interventions:**  Home visit Telephone care  Personal care assessment and treatment plans  Medication reconciliation –  add the pharmacist |
| **Document collection** | Do you have documents that will help me that might help me better understand these transitions of care?  Is there anything else you could share with me that might help? |  |  |
| **Unicorn wishes & wind down** | Is there anything else you could share with me that might help me better understand these transitions of care? What should I have asked about?  Is there anyone else you think I should interview for this project? Do you have any questions for me? |  |  |
