# Supplementary material for: Contextual Factors Affecting Continuity of Follow-Up Care After Hospital Discharge for Patients with Chronic Diseases in Sudan: A Qualitative Study with Causal Loop Diagram Insights
Source: Health Serv Insights. 2025 Jun 24;18:11786329251349916. doi: 10.1177/11786329251349916 (PMC12188063; doi:10.1177/11786329251349916)
Supplement: sj-docx-3-his-10.1177_11786329251349916 – Supplemental material for Contextual Factors Affecting Continuity of Follow-Up Care After Hospital Discharge for Patients with Chronic Diseases in Sudan: A Qualitative Study with Causal Loop Diagram Insights [file sj-docx-3-his-10.1177_11786329251349916.docx]

**Table: Quotations of the participants relevant to general themes**

| **Variable** | **Quotation** | **Participants** |
| --- | --- | --- |
| Quality of the patient's counseling sessions | 1. *For special cases like a patient on warfarin who needs regular investigation and follow-up we write the instructions in Arabic to make sure the patient can understand the warning sign, also patients who are discharged by medical devices, we ask the patient to repeat what we said to ensure understanding.* | **P8** |
|  | 1. *We have a system failure, for example, the is a patient who came all over the way from a faraway state the patient is diagnosed correctly, and their family requested to be transferred to Khartoum hospital. We can save this unnecessary transfer through proper counseling.* | **P1** |
|  | 1. *We do not have a follow-up in PHC, we see patients in the hospital after they develop the complication* | **P2** |
|  | 1. *Communication is affected by the patient’s conditions: deaf or semi-deaf, confused, elderly* | **P1** |
|  | 1. *There is bad counseling bad communication, and no clear system for the patient after discharge* | **P5** |
|  | 1. *Bad counseling, it might be that the ER is very busy* | **P1** |
|  | 1. *Communication is affected by the environment surrounding us, good counseling needs to give examples and simplify the information to the context of the patient* | **P5** |
|  | 1. *We have a patient who receives very bad counseling, I met patients who have a very serious disease, and they are not aware of anything about their condition* | **P3** |
|  | 1. *the number of doctors affects the communication with the patients, we usually don’t have enough time to provide the proper counseling* | **P5** |
| Follow-up after hospital discharge | 1. *A lot of the patients do not come back for follow-up unless there is a complication, and we might need to readmit the patient from the outpatient clinic* | **P3** |
| PHC service accessibility | 1. *The patient came to the hospital for services that can be provided in the PHC and this led to a wastage of resources, when of the main cause that the patient finds this service in the hospital for free in comparison if they went to the PHC where they pay and on the same time might not find the service available* | **P16** |
|  | 1. *There are a lot of tasks and activities the patients do in hospital that should be done in PHC, this leads to a waste of resources at the level of hospital and the PHC* | **P16** |
|  | 1. *The fundamental challenges to establishing effective primary care provision are predominantly having a suitably qualified workforce; adequate financing; quality assurance; ensuring patient safety* | **P12** |
| Sustainable quality improvement activities | 1. *The concept of the quality improvement is not well known for a lot of the stakeholders, and we usually work to inform the stakeholder about the important of the quality improvements program and we face the turnover, and we need to work again to the new stakeholder or politics* | **P12** |
|  | 1. *Quality improvement directorate in FMOH role is to improve the quality of the services provided in the PHC, when we supervise, feedback report should be taken into action at the state and district level, here it shows the poor accountability at this level* | **P12** |
|  | 1. *Everything needs total restructure* | **P4** |
|  | 1. *We have total system failure* | **P1** |
|  | 1. *The health problem needs radical solution for everything* | **P3** |
|  | 1. *The main obstacle we are facing is the mindset of the person* | **P13** |
|  | 1. *This situation is very difficult, patient education is not easy I think we should start by children.* | **P3** |
|  | 1. *I think patient the escape from the hospital during the hospital admission and they prefer to die at home than being in the hospital with this situation* | **P5** |
| Family involvement | 1. *we have language barrier for communicating with the patient and his family specially for the patient who come from rural are and speak local dialect* | **P7** |
|  | 1. *We include the family in the education because the patient a lot of the time is not educated or elderly, or stroke patients* | **P9** |
|  | 1. *I can’t say if the co- patient facilitates the management or complicate it, this is literally depending on their literacy and level of education.* | **P4** |
|  | 1. *Some family goes to other direction, so they put their own concepts and miss understanding to the patients. Some time they have bad influence on the patient* | **P3** |
|  | 1. *Family involvement is important because economically the patients depend usually on their families so they should be aware about what we are doing, they are interested to know about the cost of hospital admission, the need for rehabilitation and the prognosis of the disease.* | **P4** |
|  | 1. *Some of the patients are not educated, they are not fully understanding what we are say so we make sure the co-patients at least understand the discharge instructions* | **P3** |
|  | 1. *Counseling is challenged by the huge number of relatives and a lot of time we don’t understand who the close relative is* | **P5** |
| Political instability | 1. *Sustainability of any improvement program depend on the political influence and the decision, manager mange his work* | **P15** |
|  | 1. *We need to make compromise when at the end has it is influence on the quality of the services, we provide* | **P12** |
|  | 1. *the authority of the minister of health is affect by the politics and it happened again at the time when they adopt the emergency at the time of COVID* | **P15** |
|  | 1. *Politics has influence on the PHC expansion they decision to build a PHC they change it to hospital based on political influence and they might change the site of the PHC* | **P14** |
|  | 1. *These circumstances make that they have a very big influence pf politics on the any improvement activity (political commitment)* | **P12** |
| Infrastructure and health facility premises | 1. *There is a lack of basic hospital equipment (emergency drug and emergency equipment) we are not able to think about other improvement we are just here to make sure the basic are available.* | **P17** |
|  | 1. *Improvement of the infrastructure is affected by inflation,* | **P14** |
|  | 1. *inflation affect our plan for improvement, budget, salary, infrastructure* | **P14** |
|  | 1. *Infrastructure and disorganized work (medical records, emergency drug and equipment’s) in hospital frustrate us and affect mental well being* | **P5** |
|  | 1. *Implementation of policies is depending on, personal attitude, capacity, human resource and infrastructure* | **P16** |
|  | 1. *Implementation of referral system need infrastructure firstly we need ambulances.* | **P11** |
| Healthcare provider's turnover | 1. *HR faced by a high turnover due to the doctor’s migration, it has happened that our department had seven doctors left on the same time* | **P15** |
|  | 1. *Health worker (doctors) feel stressed and exhausted, because we do thing unrelated to our job description, wasting our time mainly in unorganized hospital management* | **P5** |
|  | 1. *The education that the registrar and the consultant provide us matter to us and our learning process.* | **P8** |
|  | 1. *Providing home visits is restricted by the human recourse issue* | **P12** |
|  | 1. *We don’t have applied retention policy, we train health care staff, and we lose them due to the high turnover, trained staff will leave the job to better situation.* | **P12** |
|  | 1. *Low wages salary is one of the causes of the turnover* | **P16** |
|  | 1. *High managerial job is not secure job, you might be transferred to another place suddenly.* | **P15** |
|  | 1. *We don’t build experience; I am working here for 7months now and I might leave at any time. Since I worked in this sector, I am ready at any moment to leave the position* | **P15** |
|  | 1. *Turnover is related to the political issues mainly when to comes to high level mangers* | **P15** |
| Motivation and work engagement of healthcare providers | 1. *These factors lead to more consumption of our time and energy which lead to less concentration and motivation in the patient’s care.* | **P5** |
|  | 1. *Education has a clear plan from senior to us as a house officer, we will have more information and provide more to the patients* | **P9** |
|  | 1. *we are drained by the other issue that waste our time and energy for example searching for patients’ kits and other thing does not part of our role.* | **P5** |
|  | 1. *The physical infrastructure, hospital hygiene affects our wellbeing.* | **P6** |
|  | 1. *We don’t feel safe sometime, co patient blame us for negligence in the hospital and they fight with us some time. There is no rule to protect the doctors* | **P7** |
| Information management system | 1. *We use paper usually we use yellow card, and we write the patient diagnosis and the basic information and what they were received in the hospital. This card is a tool for communication between the doctors and we write this information in English.* | **P3** |
|  | 1. *In the PHC we don’t have proper recording system. We use notebook to keep the patient base line readings and the follow-up notes, in fact we ask the patients to buy these notebook* | **P10** |
|  | 1. *This card saves the patient right and inform us that they have been admitted here in the hospital. Also, it saves it if the patient meets another doctor in the outpatient.* | **P9** |
|  | 1. *Patients bring their card when they are come to the outpatient’s clinic. This is the only source of information for the patient’s history* | **P9** |
|  | 1. *Even patients recording office have very poor recording system they do not write the patient diagnosis, just the name of the patient and the name of their doctor* | **P10** |
| Team harmony | 1. *We don’t depend on clinical pharmacist; I depend on my staff.* | **P2** |
|  | 1. *Clinical pharmacist depends on the book, I have experience that I share with my join doctors, unless this clinical pharmacist work with me* | **P2** |
|  | 1. *I have experience that the pharmacist adjusts the patient dose without they communicate with us, like this attitude pissed off* | **P2** |
|  | 1. *Teamwork and interdisciplinary approach need the team leader welling and their network* | **P12** |
|  | 1. *I don’t see any kind of collaboration between nursing staff and the doctors in patients counseling, it would be better if they focus in their work and provided the doses in the right time.* | **P4** |
| Personal network | 1. *We as staff are cooperative with each other’s, doctors do their best and these problems are out of their control* | **P4** |
|  | 1. *I depend on my personal network, I know most of the consultant in the states so I refer my patients to someone I know, if there is no consultant that I know a consultant in that state, I just write them referral letter to any Physician to continue their care* | **P1** |
|  | 1. *The communication between the doctors goes well specially if we need advice and consultation form specialist on case usually, we send the patient with a letter and they feedback us by the consultation and advices, some of the even call and ask for the patient investigation and we communicate this information through the WhatsApp* | **P2** |
|  | 1. *we face real issue when we need to refer the patient to be under their supervision, it is usually not easy to get the patient acceptance, due to lack of vacancy* | **P4** |
|  | 1. *Refereeing patient to another hospital; is a real problem, we try to reach out the consultant and discuss the case with them, because it is usually doesn’t work if we send them, medical directors of the hospital usually argue we don’t have any vacancy* | **P4** |
|  | 1. *Despite the challenges that we face in patients care the communication between us, the doctors are very good, we communicate and discuss the patients care through phone call or face to face* | **P6** |
|  | 1. *I have another initiative, it is called -------: it is mobile app, we are connecting doctors with the patients virtually and we plan in the future to add home visit service.* | **P1** |
|  | 1. *As doctors, we meet a lot of patients who are financially struggling, some time we collect money from us and try to help them may be do some expensive important investigation or help in transportation.* | **P2** |
|  | 1. *Two house officers worked with me, they financially supported a patient who is coming from far away state, and they take the responsibility of travel with them to make sure he went back to his city safe* | **P2** |
| telecommunication technology | 1. *The education level of the patient also affects the usage of the service from example if you give them a contact number, they are not aware when they should call and what case they should call for.* | **P6** |
|  | 1. *We give our phone to the serious cases that need monitoring, for example case of DVT and need monitoring and INR, also we give out to contact to some patients who need follow-up and live in faraway state. Also, if the patient health literacy is low you want to ensure that they are understanding what you have said and you know that they don’t have any other health access, investigation result and adjust the dose* | **P3** |
|  | 1. *It is some time part of the patients discharge plan, we discharge the patients and then we need to adjust the dose and the patients doesn’t need to be admitted for this purpose. This is not part of the hospital policy* | **P4** |
|  | 1. *We give our contact tot specific patient who needs our support for different reasons, type of the condition, medication they us e and the health literacy* | **P8** |
|  | 1. *This is voluntary work of the doctors and part of the hospital policy* | **P4** |
|  | 1. *Telemedicine is possible and applicable* | **P1** |
|  | 1. *Some patients they send their investigation through WhatsApp. They call us because some time if the clinical doesn’t work (strike) so, calls save their time and money.* | **P3** |
|  | 1. *The house office who responsible of the case share their name with the co patient during the hospital discharge to facilitate the management during the hospital admission* | **P6** |
|  | 1. *A lot of the doctors suggest the use of the phone by the patients as solution that save patients time and money.* | **P3** |
|  | 1. *The issue that the patients have our personal contact number when you finish you shift in the department the patient loss the contact to the hospital. This work needs to be systematic and organized.* | **P6** |
|  | 1. *There was call center for the referral system, this call system helped us to identify the defect in the ICU, nursing care and the CCU* | **P3** |
|  | 1. *We have experience with the call center to manage case of COVID at home and it went well.* | **P6** |
| Functioning referral system | 1. *The health care providers stated that, there is no clear referral health system, after patient discharge we don’t know where the patients can go. We usually instruct the patients, so tell them to go to the nearest hospital if they develop any complications. We are concern that they might not find any functioning PHC.* | **P1** |
| Governance and accountability system | 1. *The role of FMOH is supervision and planning are the role of the state ministry of health. The state ministry of health has autonomy, and the same time should be accountable for the feedback of the supervision from the FMOH, which usually does not happen Sudanese attitude, they don’t follow roles and policies at the level of the stakeholder* | **P15** |
|  | 1. *The governing role of the FMOH doesn’t allow the authorities to change anything, they might respect him, but these authorities are at the local level. So, any project’s success depends on the personal interest of those who have been hired for this position, priorities, and understanding of the issue.* | **P15** |
|  | 1. *The power and the authority that has been given to the minister during the transition period 2019 – 2021, he was able to appoint state ministers and general directors at the level of the state.* | **P12** |
|  | 1. *In 2015, the referral system was a priority in the FMOH, there were two committees, at the higher level, which used to meet every month. Another one at the local and exudative level which they used to meet every week. These meeting recommendations are priorities and there was regular supervision and accountability at the level. These meetings report the causes of unnecessary referrals and wrong referrals.* | **P16** |
|  | 1. *There is no clear cut which cases can be managed in general medicine ward, and which need to be referred, they argue usual is about unnecessary referral* | **P1** |
|  | 1. *Policies are not implemented without personal network, and we saw that in Aljazeera state where the social connection is bigger than the political influence you can see that they work in harmony to the opposite to other state where political influence is major* | **P12** |
|  | 1. *The policy implementation depends on the personal preference if any turnover happen this will lead to stop of the implementation* | **P12** |
|  | 1. *The role of the quality directorate is to ensure safe transfer of the patients from on hospital to another* | **P12** |
|  | 1. *Health insurance policy should ensure that the patient come through the PHC, but this cannot be implemented because the PHC has to ensure that they are functioning, taking into consideration the insurance coverage is very low* | **P16** |
|  | 1. *Effective referral system will help in effect health service planning if you know how much of the case are coming from which states* | **P16** |
|  | 1. *Accountability can be improved by compliant box, involve the end user in the accountability and reporting effect in referral system* | **P16** |
|  | 1. *Policies usual either not well written, or if written not implemented* | **P16** |
|  | 1. *Protocol of case management are also needed to ensure the good referral system* | **P12** |
|  | 1. *Referral policies are affected by the ability to implement policies and policies implementation tools.* | **P13** |
